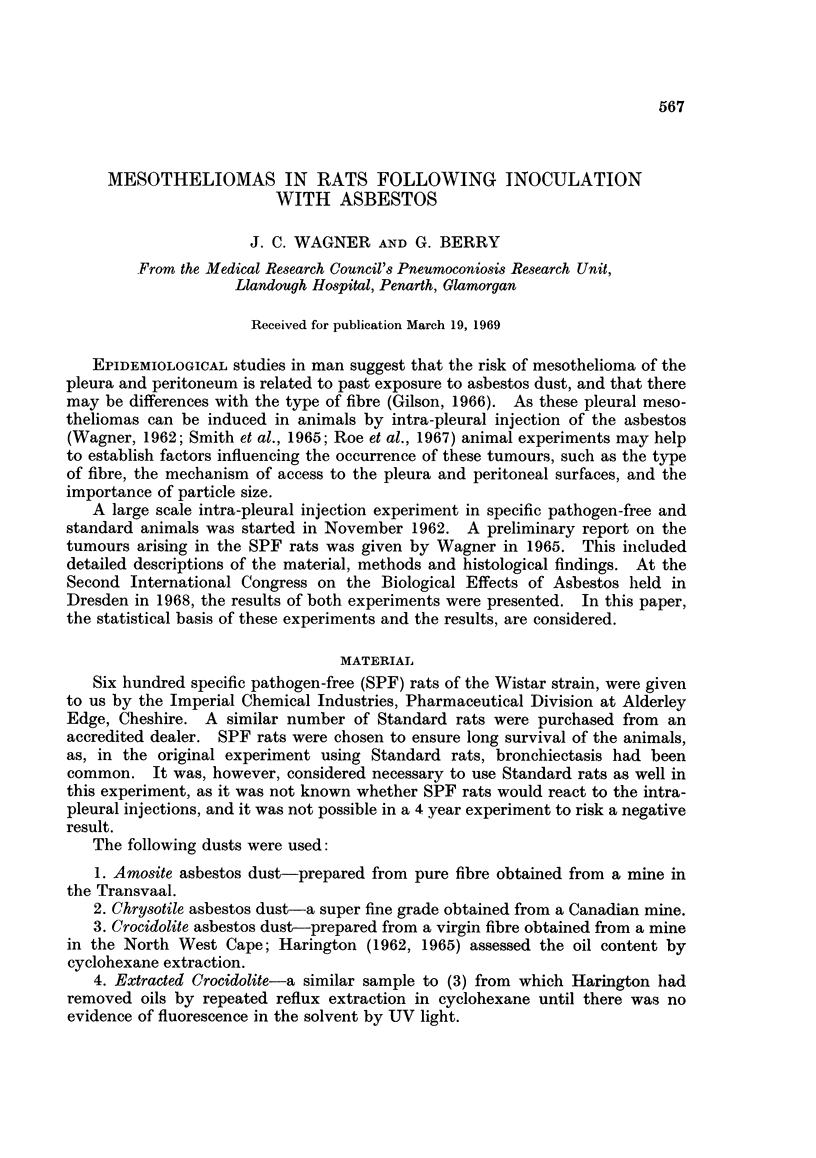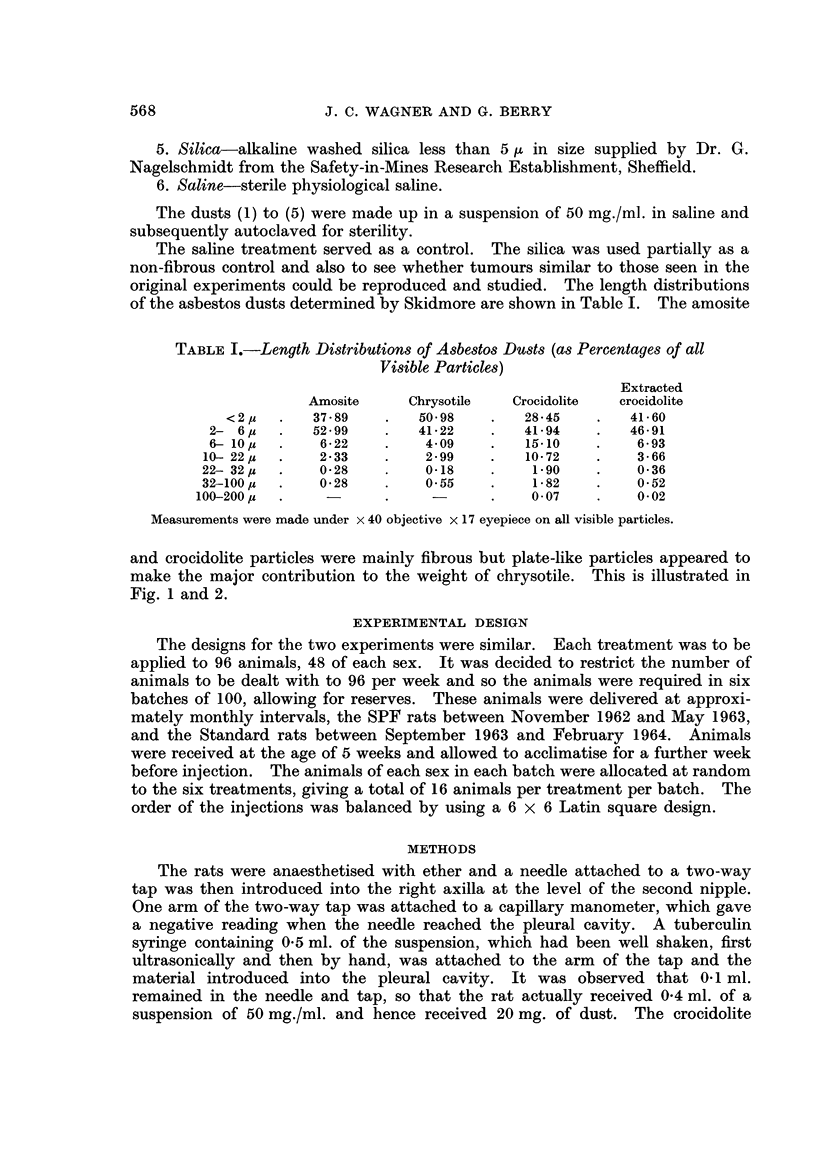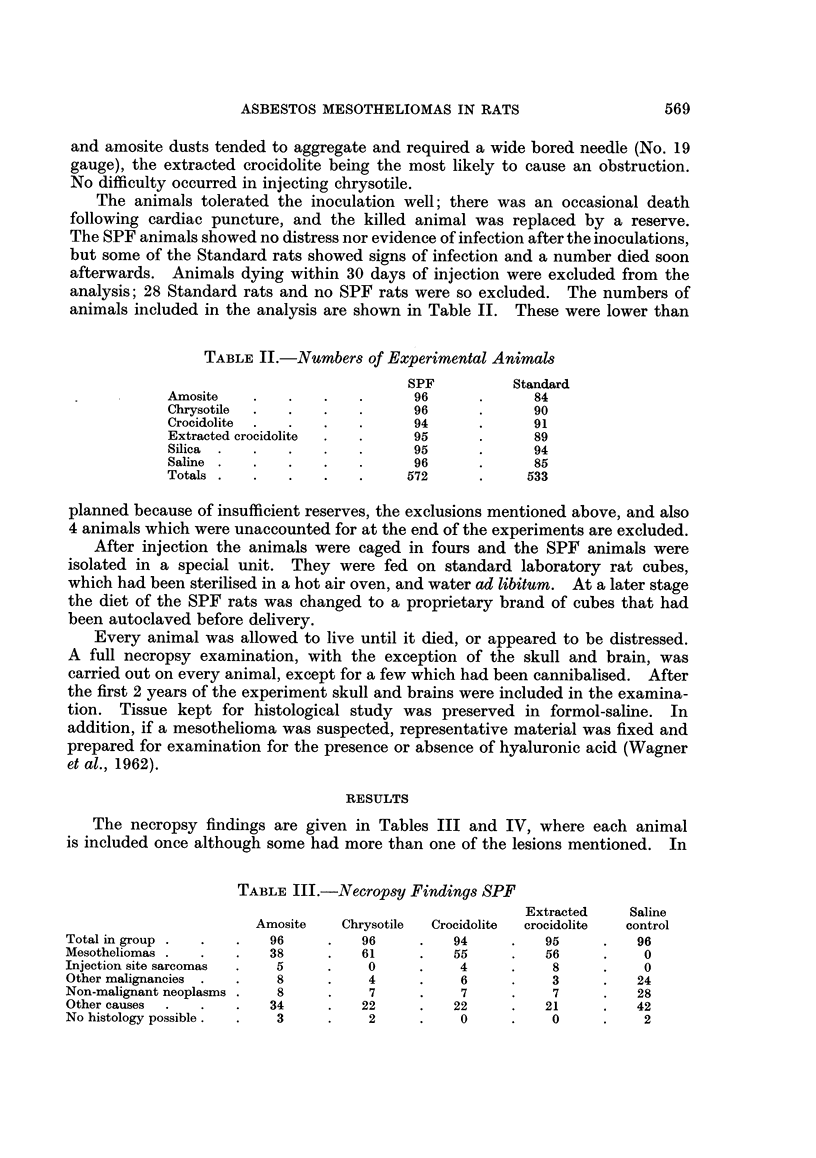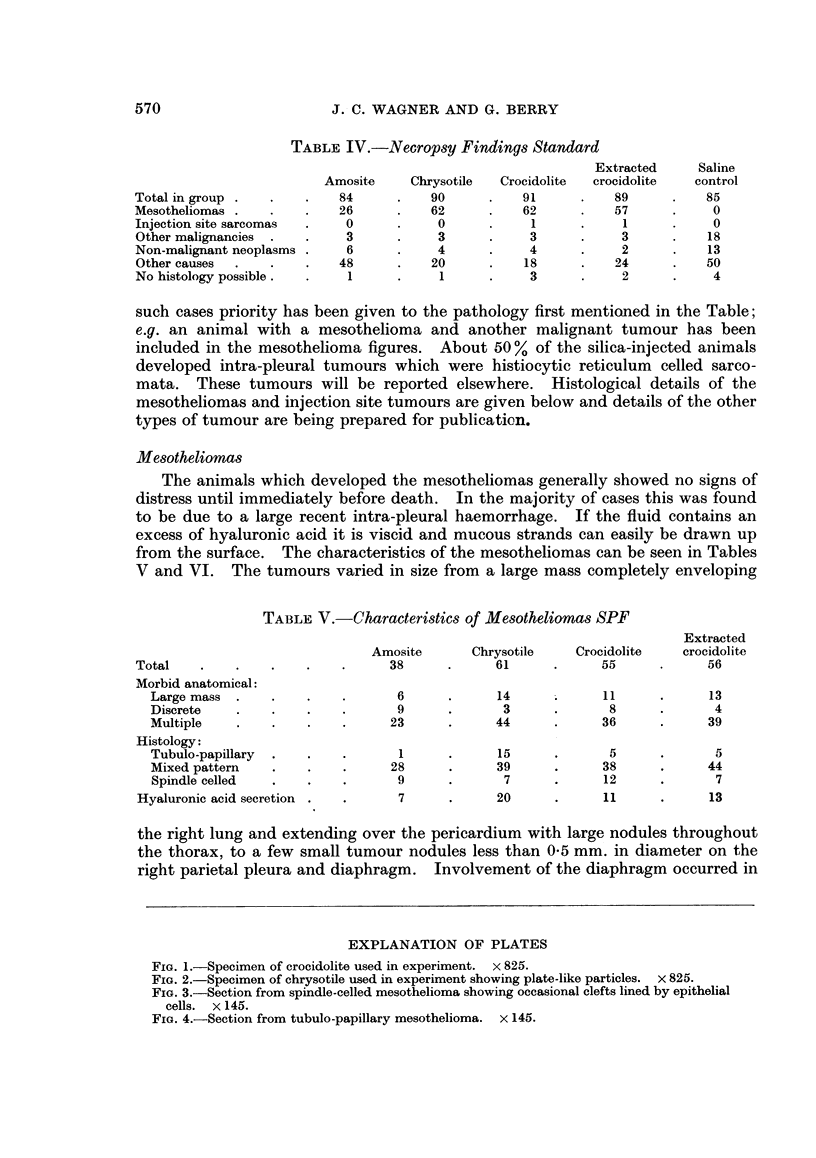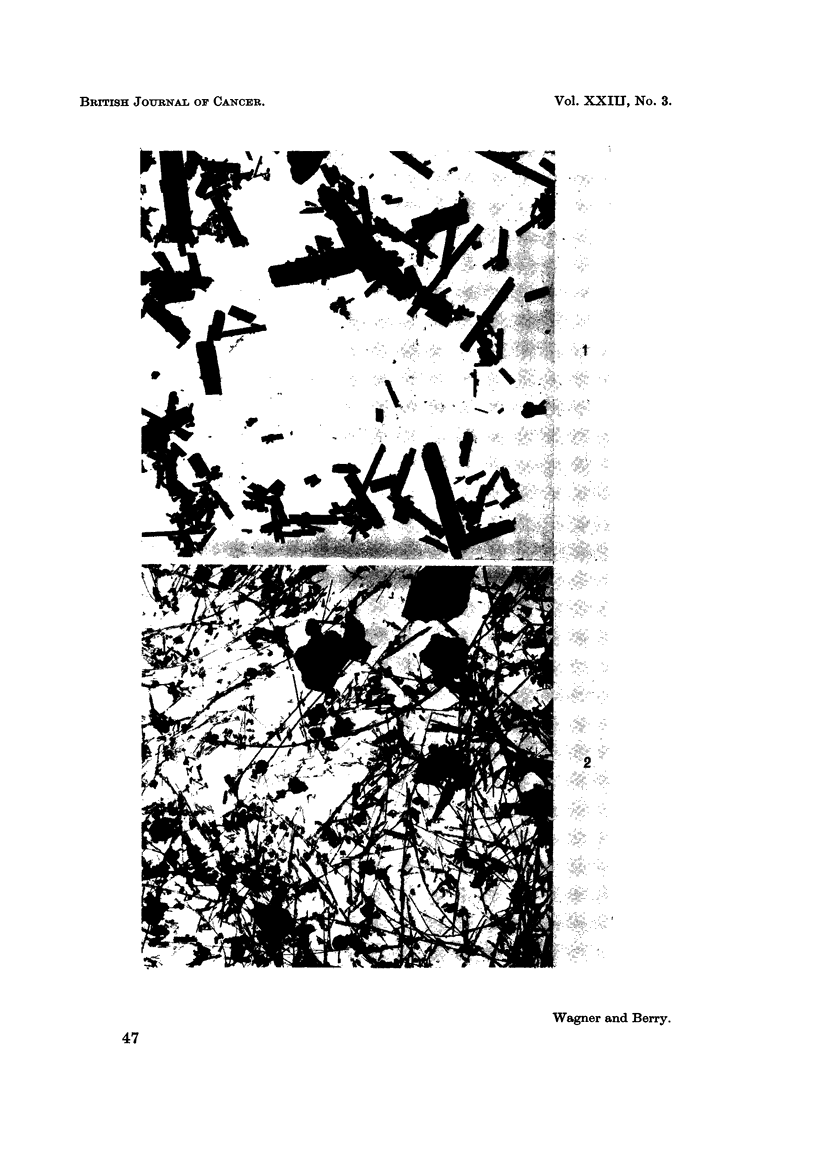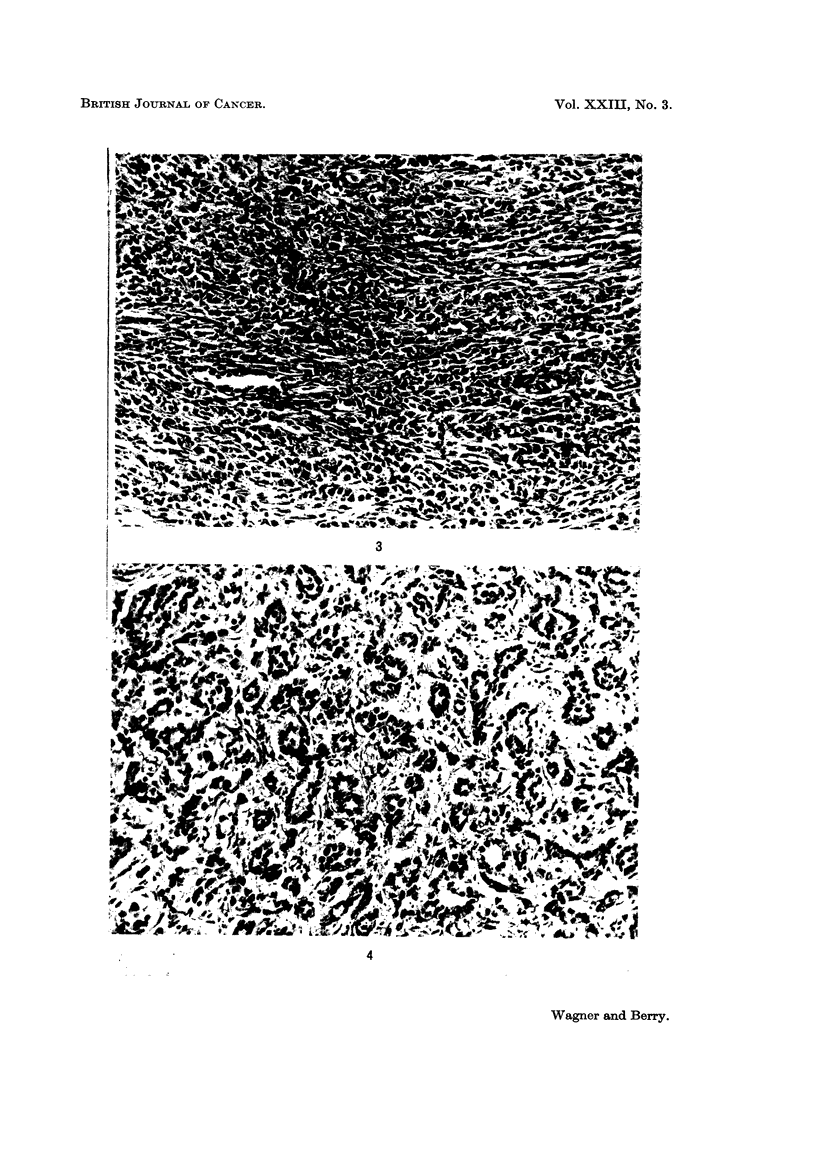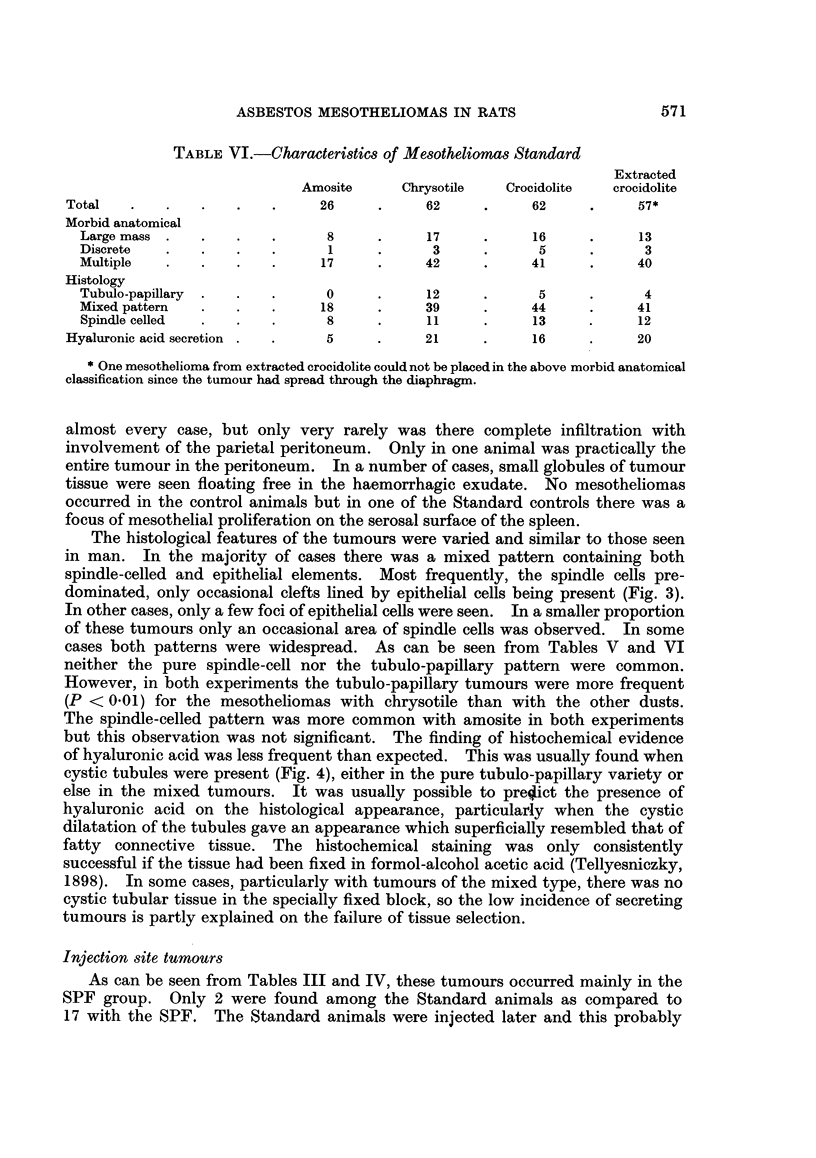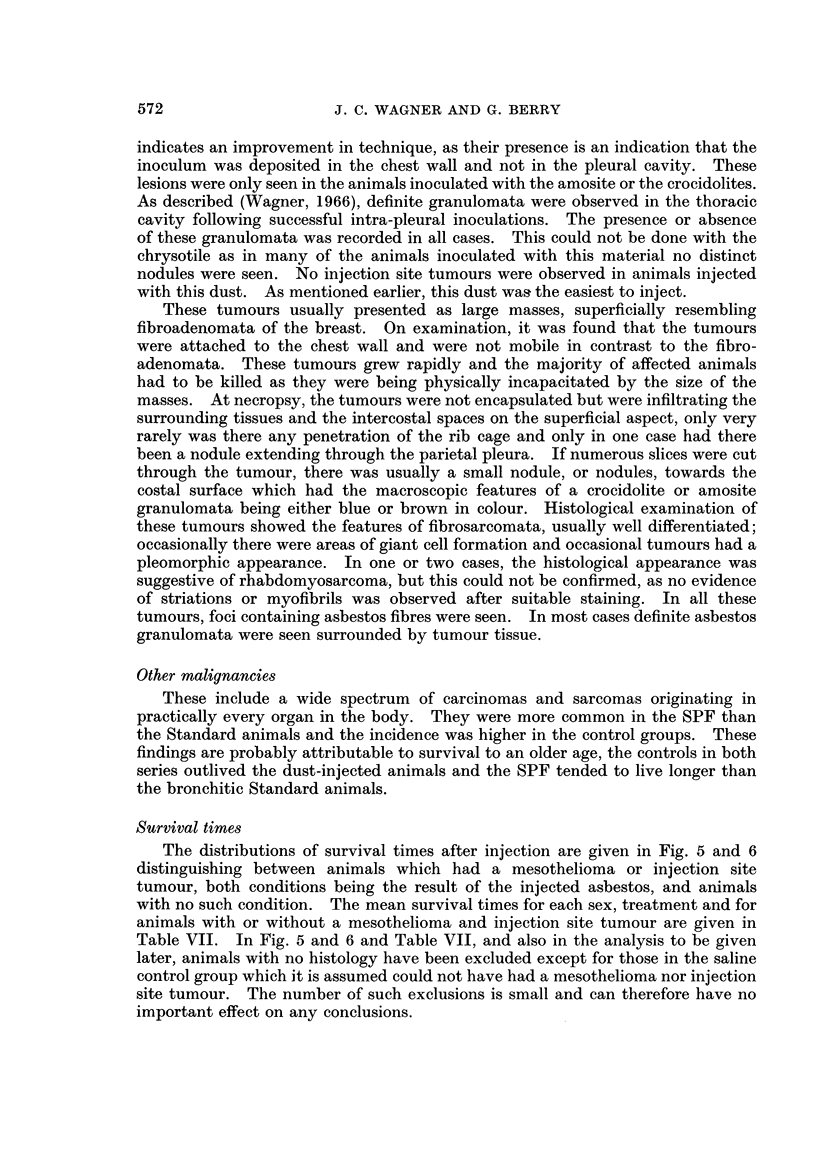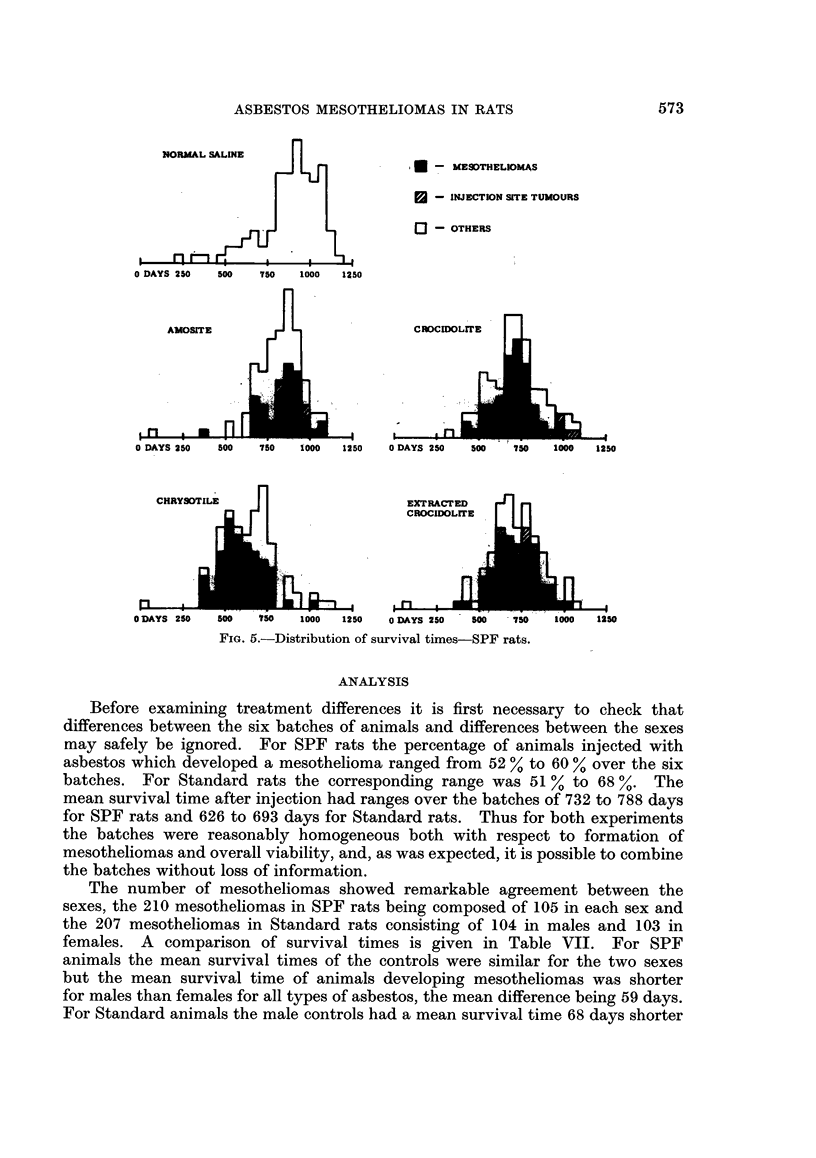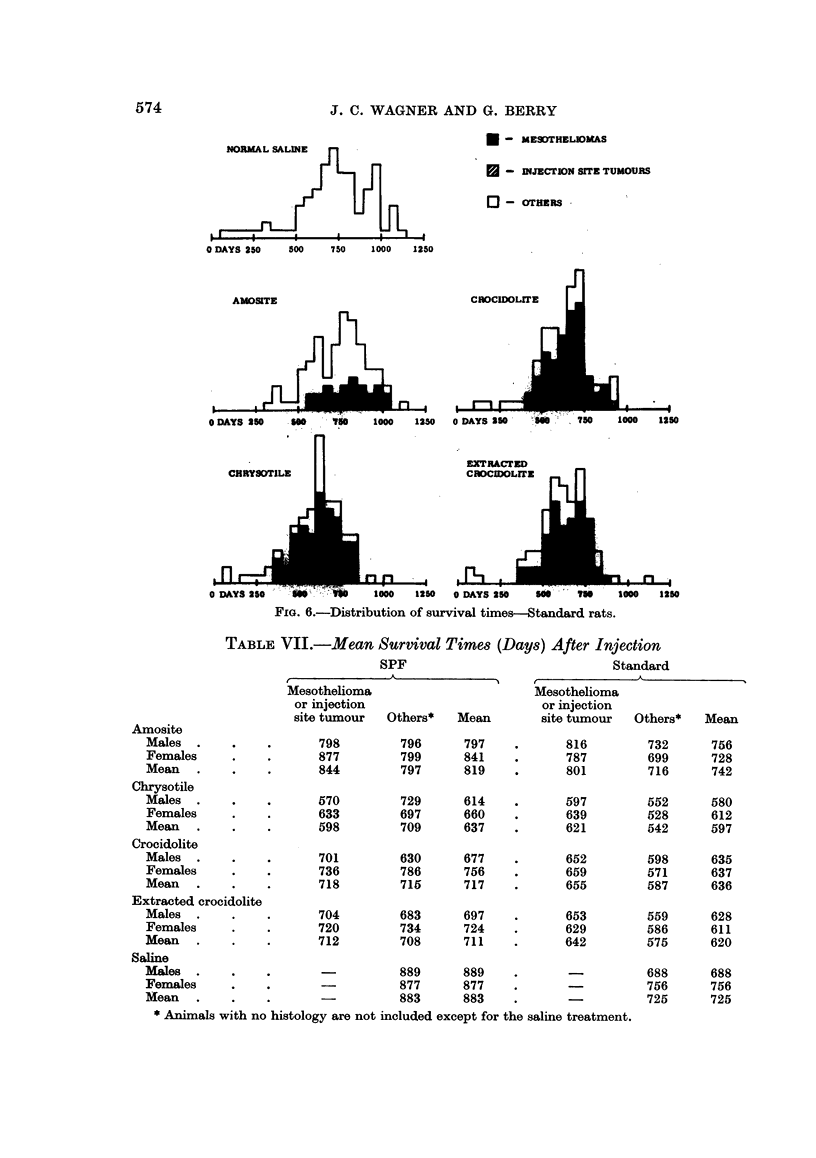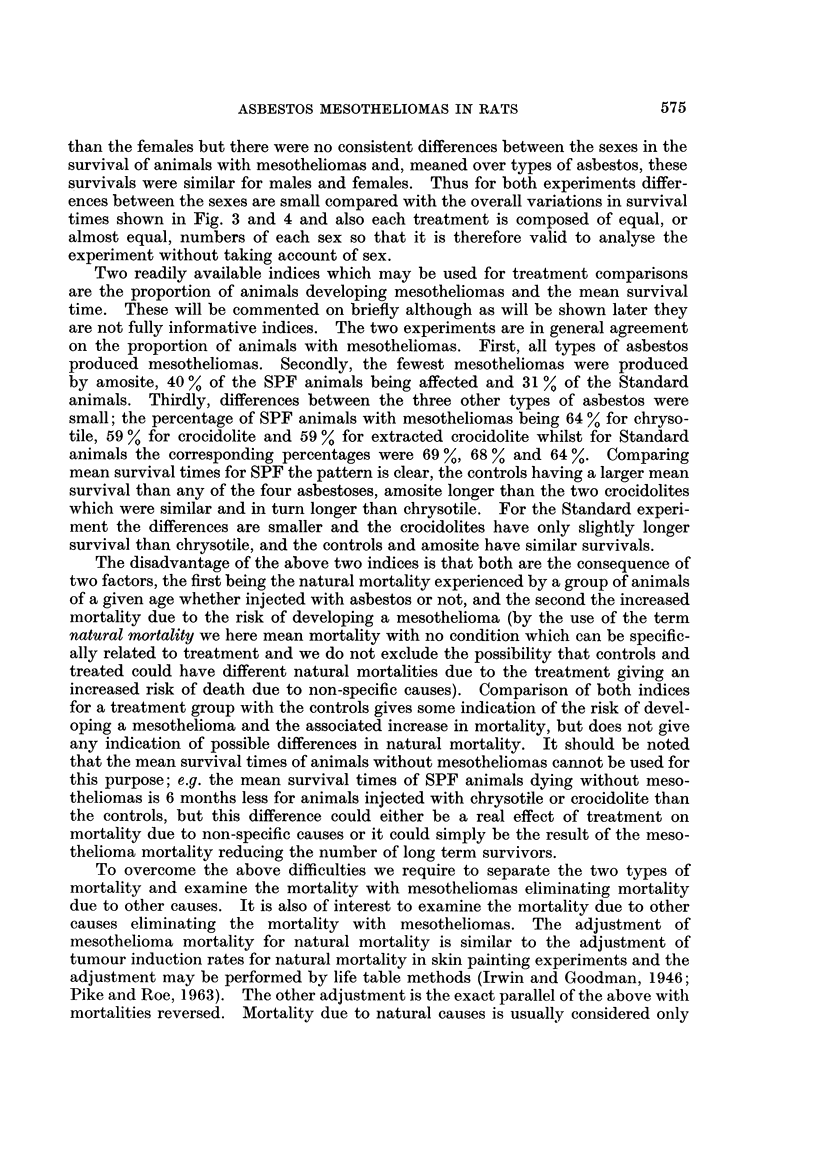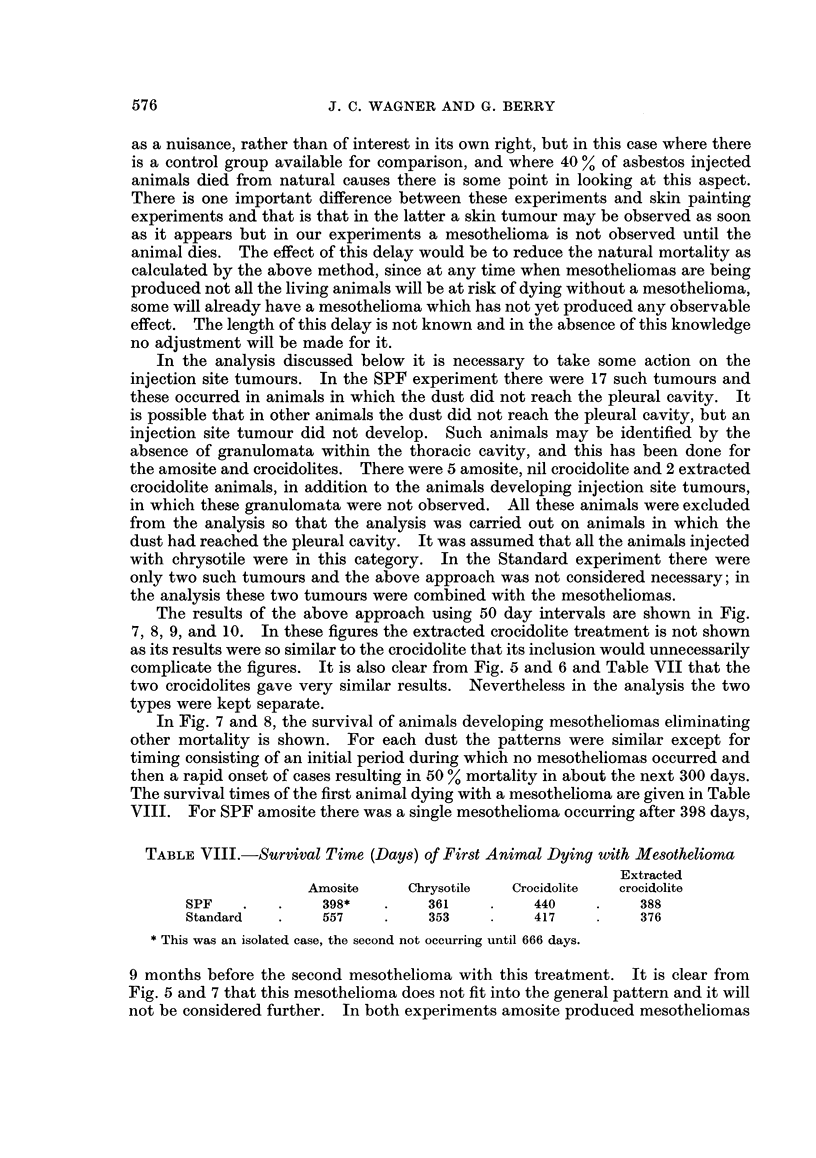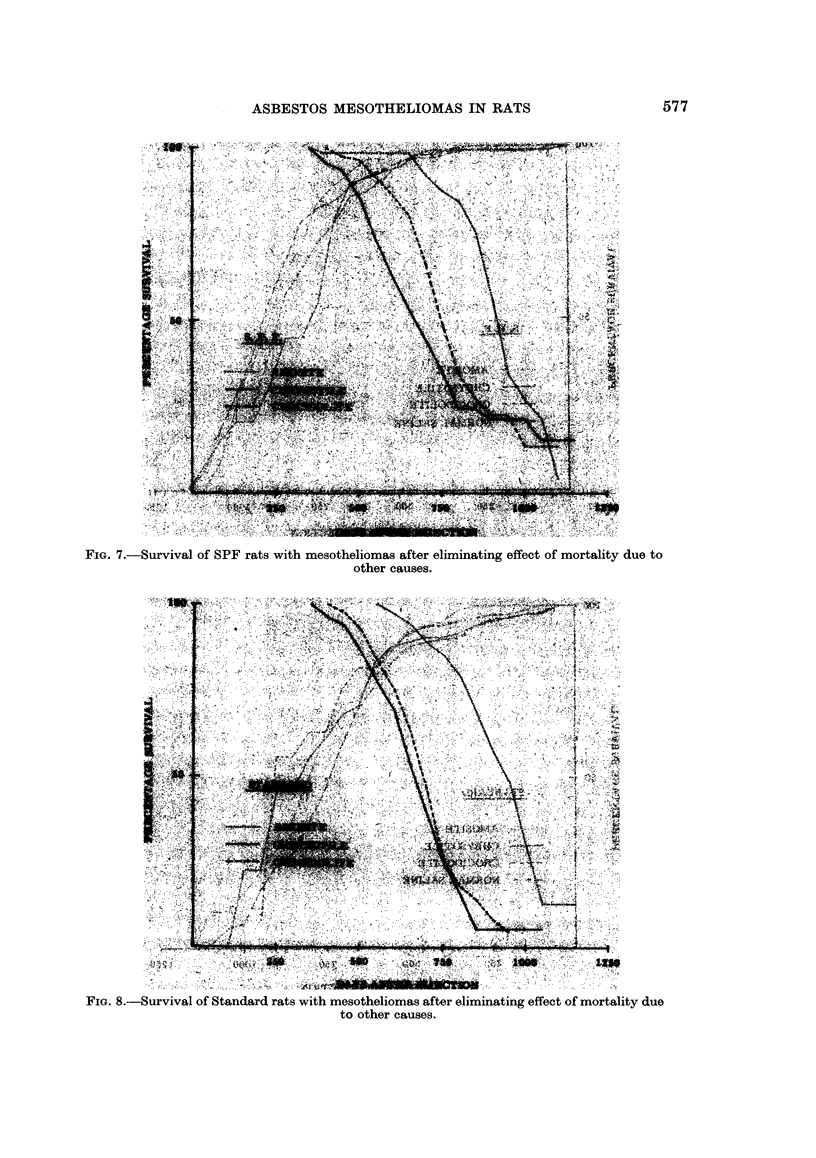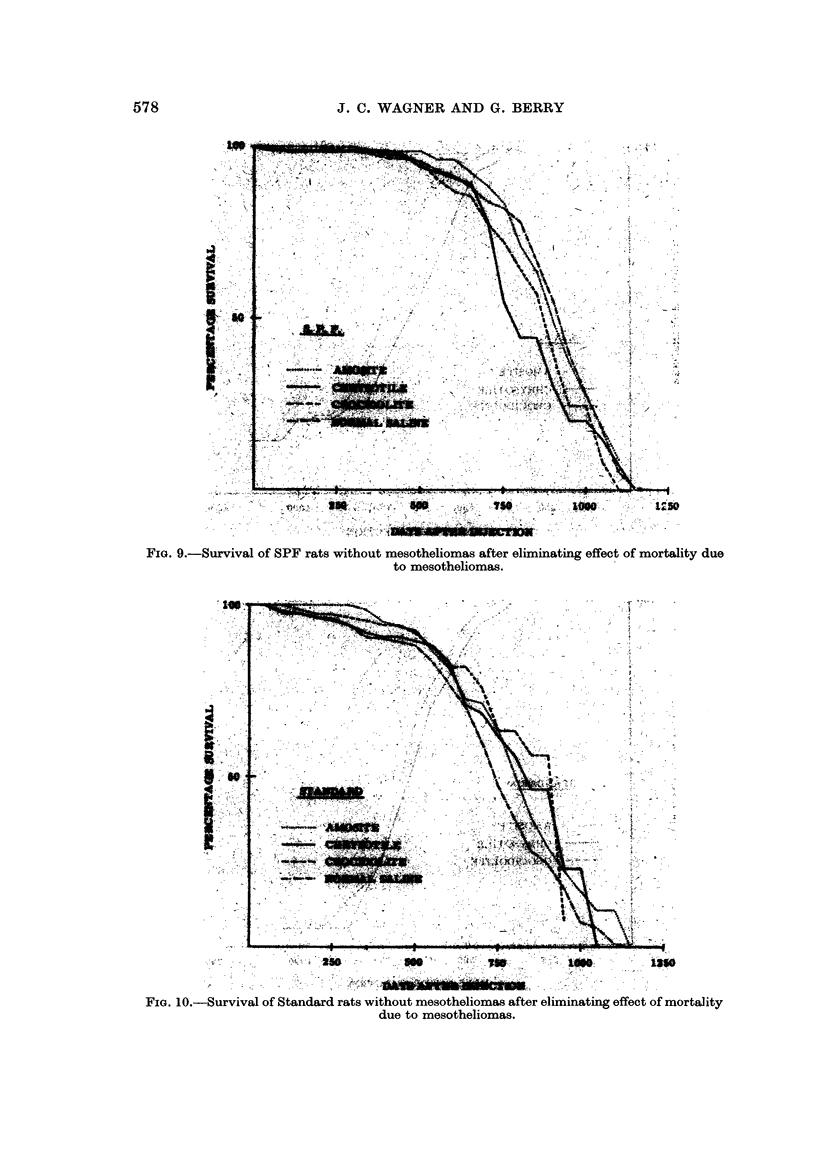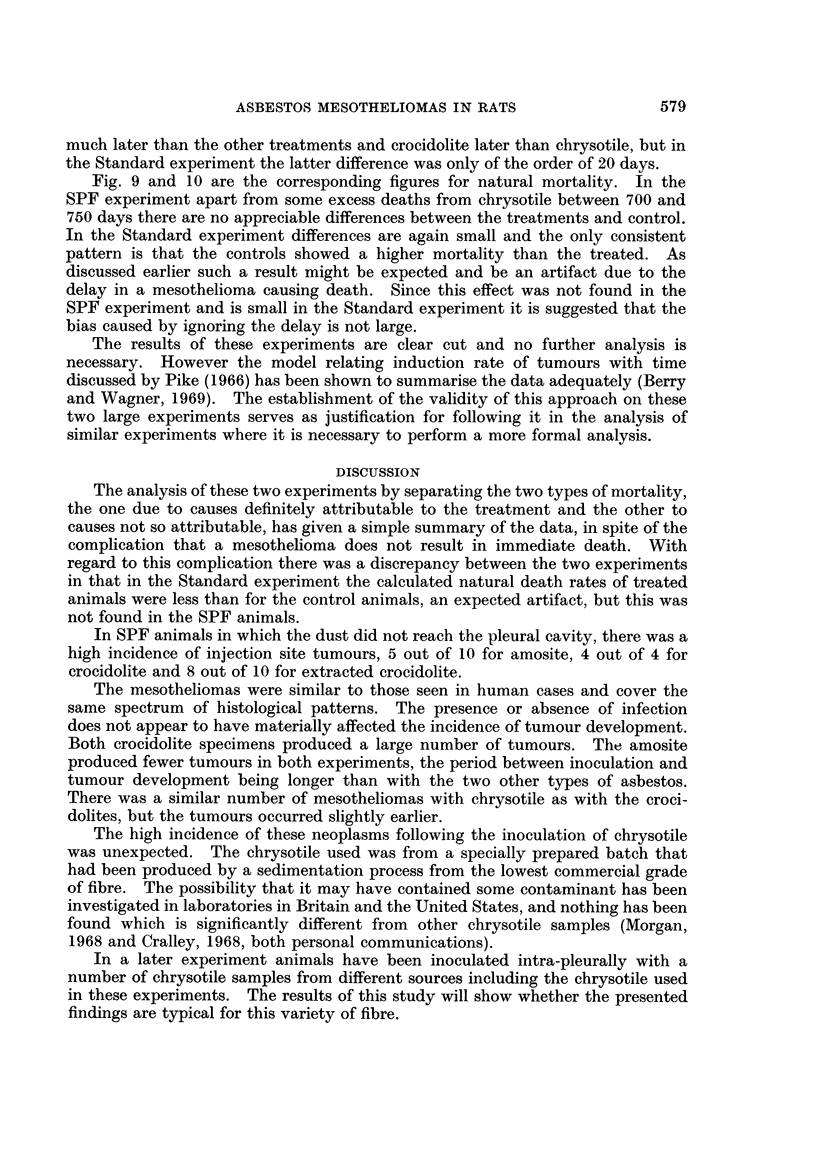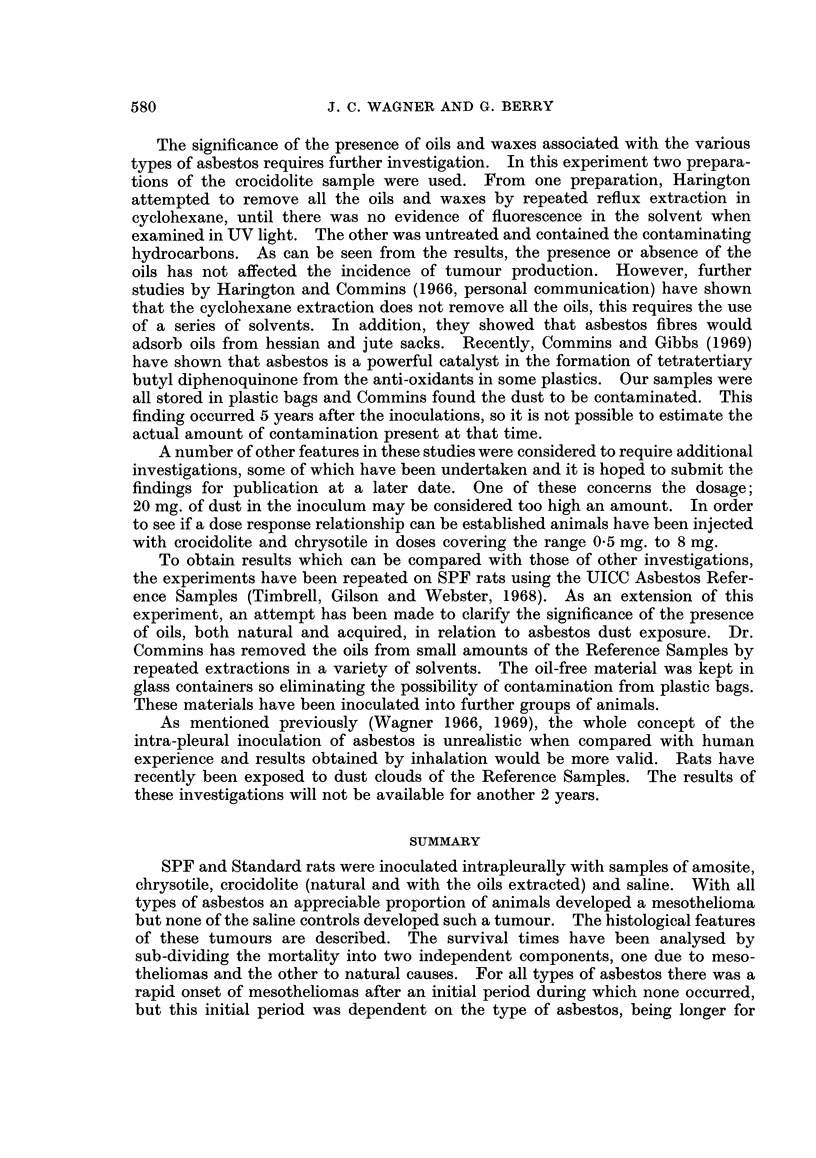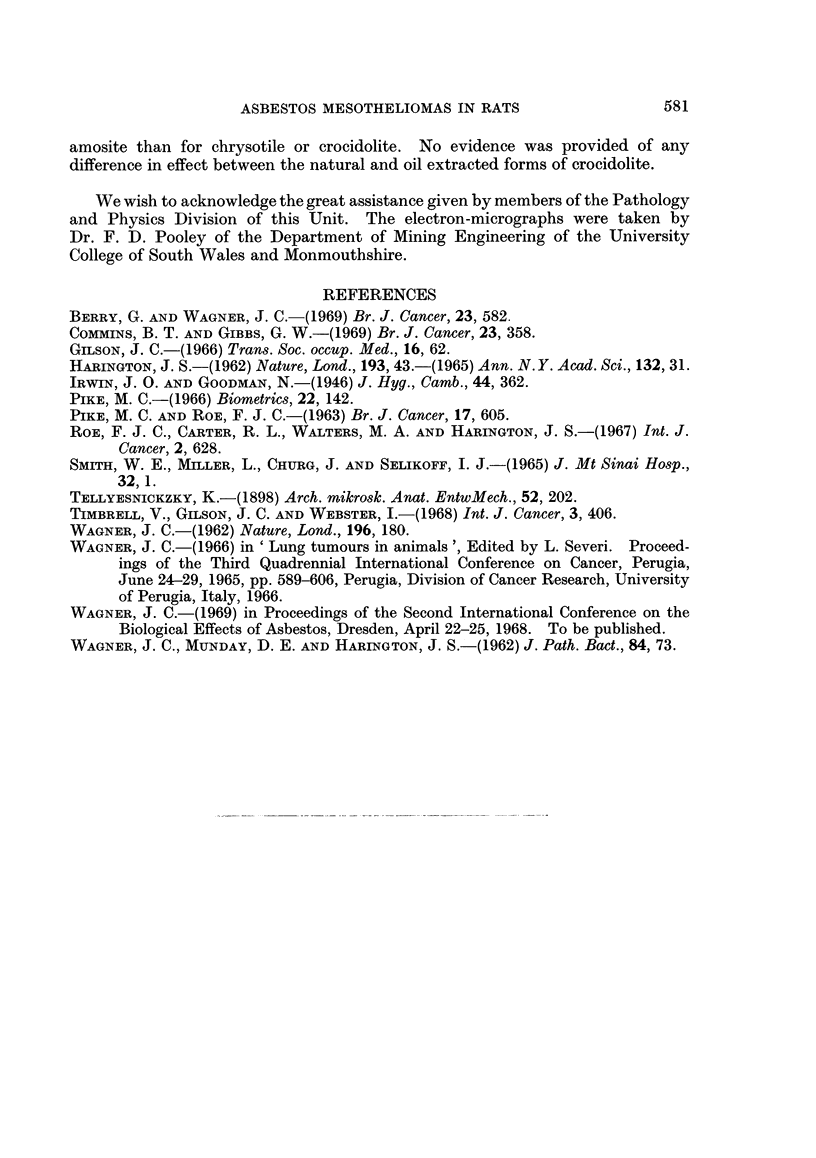# Mesotheliomas in rats following inoculation with asbestos.

**DOI:** 10.1038/bjc.1969.70

**Published:** 1969-09

**Authors:** J. C. Wagner, G. Berry

## Abstract

**Images:**


### MESOTHELIOMAS IN RATS FOLLOWING INOCULATION WITH ASBESTOS

J. C. WAGNER AND G. BERRY

*From the Medical Research Council's Pneumoconiosis Research Unit, Llandough Hospital, Penarth, Glamorgan*

Received for publication March 19, 1969

EPIDEMIOLOGICAL studies in man suggest that the risk of mesothelioma of the pleura and peritoneum is related to past exposure to asbestos dust, and that there may be differences with the type of fibre (Gilson, 1966). As these pleural mesotheliomas can be induced in animals by intra-pleural injection of the asbestos (Wagner, 1962; Smith *et al.*, 1965; Roe *et al.*, 1967) animal experiments may help to establish factors influencing the occurrence of these tumours, such as the type of fibre, the mechanism of access to the pleura and peritoneal surfaces, and the importance of particle size.

A large scale intra-pleural injection experiment in specific pathogen-free and standard animals was started in November 1962. A preliminary report on the tumours arising in the SPF rats was given by Wagner in 1965. This included detailed descriptions of the material, methods and histological findings. At the Second International Congress on the Biological Effects of Asbestos held in Dresden in 1968, the results of both experiments were presented. In this paper, the statistical basis of these experiments and the results, are considered.

### MATERIAL

Six hundred specific pathogen-free (SPF) rats of the Wistar strain, were given to us by the Imperial Chemical Industries, Pharmaceutical Division at Alderley Edge, Cheshire. A similar number of Standard rats were purchased from an accredited dealer. SPF rats were chosen to ensure long survival of the animals, as, in the original experiment using Standard rats, bronchiectasis had been common. It was, however, considered necessary to use Standard rats as well in this experiment, as it was not known whether SPF rats would react to the intra-pleural injections, and it was not possible in a 4 year experiment to risk a negative result.

The following dusts were used:

1. *Amosite* asbestos dust—prepared from pure fibre obtained from a mine in the Transvaal.
2. *Chrysotile* asbestos dust—a super fine grade obtained from a Canadian mine.
3. *Crocidolite* asbestos dust—prepared from a virgin fibre obtained from a mine in the North West Cape; Harington (1962, 1965) assessed the oil content by cyclohexane extraction.
4. *Extracted Crocidolite*—a similar sample to (3) from which Harington had removed oils by repeated reflux extraction in cyclohexane until there was no evidence of fluorescence in the solvent by UV light.

5. *Silica*—alkaline washed silica less than 5 μ in size supplied by Dr. G. Nagelschmidt from the Safety-in-Mines Research Establishment, Sheffield.
6. *Saline*—sterile physiological saline.

The dusts (1) to (5) were made up in a suspension of 50 mg./ml. in saline and subsequently autoclaved for sterility.

The saline treatment served as a control. The silica was used partially as a non-fibrous control and also to see whether tumours similar to those seen in the original experiments could be reproduced and studied. The length distributions of the asbestos dusts determined by Skidmore are shown in Table I. The amosite and crocidolite particles were mainly fibrous but plate-like particles appeared to make the major contribution to the weight of chrysotile. This is illustrated in Fig. 1 and 2.

TABLE I.—*Length Distributions of Asbestos Dusts (as Percentages of all Visible Particles)*

| | Amosite | Chrysotile | Crocidolite | Extracted crocidolite |
|---|---|---|---|---|
| <2 μ | 37·89 | 50·98 | 28·45 | 41·60 |
| 2– 6 μ | 52·99 | 41·22 | 41·94 | 46·91 |
| 6– 10 μ | 6·22 | 4·09 | 15·10 | 6·93 |
| 10– 22 μ | 2·33 | 2·99 | 10·72 | 3·66 |
| 22– 32 μ | 0·28 | 0·18 | 1·90 | 0·36 |
| 32–100 μ | 0·28 | 0·55 | 1·82 | 0·52 |
| 100–200 μ | — | — | 0·07 | 0·02 |

Measurements were made under ×40 objective ×17 eyepiece on all visible particles.

### EXPERIMENTAL DESIGN

The designs for the two experiments were similar. Each treatment was to be applied to 96 animals, 48 of each sex. It was decided to restrict the number of animals to be dealt with to 96 per week and so the animals were required in six batches of 100, allowing for reserves. These animals were delivered at approximately monthly intervals, the SPF rats between November 1962 and May 1963, and the Standard rats between September 1963 and February 1964. Animals were received at the age of 5 weeks and allowed to acclimatise for a further week before injection. The animals of each sex in each batch were allocated at random to the six treatments, giving a total of 16 animals per treatment per batch. The order of the injections was balanced by using a 6 × 6 Latin square design.

### METHODS

The rats were anaesthetised with ether and a needle attached to a two-way tap was then introduced into the right axilla at the level of the second nipple. One arm of the two-way tap was attached to a capillary manometer, which gave a negative reading when the needle reached the pleural cavity. A tuberculin syringe containing 0·5 ml. of the suspension, which had been well shaken, first ultrasonically and then by hand, was attached to the arm of the tap and the material introduced into the pleural cavity. It was observed that 0·1 ml. remained in the needle and tap, so that the rat actually received 0·4 ml. of a suspension of 50 mg./ml. and hence received 20 mg. of dust. The crocidolite and amosite dusts tended to aggregate and required a wide bored needle (No. 19 gauge), the extracted crocidolite being the most likely to cause an obstruction. No difficulty occurred in injecting chrysotile.

The animals tolerated the inoculation well; there was an occasional death following cardiac puncture, and the killed animal was replaced by a reserve. The SPF animals showed no distress nor evidence of infection after the inoculations, but some of the Standard rats showed signs of infection and a number died soon afterwards. Animals dying within 30 days of injection were excluded from the analysis; 28 Standard rats and no SPF rats were so excluded. The numbers of animals included in the analysis are shown in Table II. These were lower than

TABLE II.—*Numbers of Experimental Animals*

| | SPF | Standard |
|---|---|---|
| Amosite | 96 | 84 |
| Chrysotile | 96 | 90 |
| Crocidolite | 94 | 91 |
| Extracted crocidolite | 95 | 89 |
| Silica | 95 | 94 |
| Saline | 96 | 85 |
| Totals | 572 | 533 |

planned because of insufficient reserves, the exclusions mentioned above, and also 4 animals which were unaccounted for at the end of the experiments are excluded.

After injection the animals were caged in fours and the SPF animals were isolated in a special unit. They were fed on standard laboratory rat cubes, which had been sterilised in a hot air oven, and water *ad libitum*. At a later stage the diet of the SPF rats was changed to a proprietary brand of cubes that had been autoclaved before delivery.

Every animal was allowed to live until it died, or appeared to be distressed. A full necropsy examination, with the exception of the skull and brain, was carried out on every animal, except for a few which had been cannibalised. After the first 2 years of the experiment skull and brains were included in the examination. Tissue kept for histological study was preserved in formol-saline. In addition, if a mesothelioma was suspected, representative material was fixed and prepared for examination for the presence or absence of hyaluronic acid (Wagner *et al.*, 1962).

### RESULTS

The necropsy findings are given in Tables III and IV, where each animal is included once although some had more than one of the lesions mentioned. In

TABLE III.—*Necropsy Findings SPF*

| | Amosite | Chrysotile | Crocidolite | Extracted crocidolite | Saline control |
|---|---|---|---|---|---|
| Total in group | 96 | 96 | 94 | 95 | 96 |
| Mesotheliomas | 38 | 61 | 55 | 56 | 0 |
| Injection site sarcomas | 5 | 0 | 4 | 8 | 0 |
| Other malignancies | 8 | 4 | 6 | 3 | 24 |
| Non-malignant neoplasms | 8 | 7 | 7 | 7 | 28 |
| Other causes | 34 | 22 | 22 | 21 | 42 |
| No histology possible | 3 | 2 | 0 | 0 | 2 |

TABLE IV.—*Necropsy Findings Standard*

| | Amosite | Chrysotile | Crocidolite | Extracted crocidolite | Saline control |
|---|---|---|---|---|---|
| Total in group | 84 | 90 | 91 | 89 | 85 |
| Mesotheliomas | 26 | 62 | 62 | 57 | 0 |
| Injection site sarcomas | 0 | 0 | 1 | 1 | 0 |
| Other malignancies | 3 | 3 | 3 | 3 | 18 |
| Non-malignant neoplasms | 6 | 4 | 4 | 2 | 13 |
| Other causes | 48 | 20 | 18 | 24 | 50 |
| No histology possible | 1 | 1 | 3 | 2 | 4 |

such cases priority has been given to the pathology first mentioned in the Table; *e.g.* an animal with a mesothelioma and another malignant tumour has been included in the mesothelioma figures. About 50% of the silica-injected animals developed intra-pleural tumours which were histiocytic reticulum celled sarcomata. These tumours will be reported elsewhere. Histological details of the mesotheliomas and injection site tumours are given below and details of the other types of tumour are being prepared for publication.

*Mesotheliomas*

The animals which developed the mesotheliomas generally showed no signs of distress until immediately before death. In the majority of cases this was found to be due to a large recent intra-pleural haemorrhage. If the fluid contains an excess of hyaluronic acid it is viscid and mucous strands can easily be drawn up from the surface. The characteristics of the mesotheliomas can be seen in Tables V and VI. The tumours varied in size from a large mass completely enveloping the right lung and extending over the pericardium with large nodules throughout the thorax, to a few small tumour nodules less than 0·5 mm. in diameter on the right parietal pleura and diaphragm. Involvement of the diaphragm occurred in

TABLE V.—*Characteristics of Mesotheliomas SPF*

| | Amosite | Chrysotile | Crocidolite | Extracted crocidolite |
|---|---|---|---|---|
| Total | 38 | 61 | 55 | 56 |
| Morbid anatomical: | | | | |
| Large mass | 6 | 14 | 11 | 13 |
| Discrete | 9 | 3 | 8 | 4 |
| Multiple | 23 | 44 | 36 | 39 |
| Histology: | | | | |
| Tubulo-papillary | 1 | 15 | 5 | 5 |
| Mixed pattern | 28 | 39 | 38 | 44 |
| Spindle celled | 9 | 7 | 12 | 7 |
| Hyaluronic acid secretion | 7 | 20 | 11 | 13 |

EXPLANATION OF PLATES

FIG. 1.—Specimen of crocidolite used in experiment. ×825.
FIG. 2.—Specimen of chrysotile used in experiment showing plate-like particles. ×825.
FIG. 3.—Section from spindle-celled mesothelioma showing occasional clefts lined by epithelial cells. ×145.
FIG. 4.—Section from tubulo-papillary mesothelioma. ×145.

1

2

Wagner and Berry.

3

4

Wagner and Berry.

TABLE VI.—*Characteristics of Mesotheliomas Standard*

| | Amosite | Chrysotile | Crocidolite | Extracted crocidolite |
|---|---|---|---|---|
| Total | 26 | 62 | 62 | 57* |
| Morbid anatomical | | | | |
| Large mass | 8 | 17 | 16 | 13 |
| Discrete | 1 | 3 | 5 | 3 |
| Multiple | 17 | 42 | 41 | 40 |
| Histology | | | | |
| Tubulo-papillary | 0 | 12 | 5 | 4 |
| Mixed pattern | 18 | 39 | 44 | 41 |
| Spindle celled | 8 | 11 | 13 | 12 |
| Hyaluronic acid secretion | 5 | 21 | 16 | 20 |

* One mesothelioma from extracted crocidolite could not be placed in the above morbid anatomical classification since the tumour had spread through the diaphragm.

almost every case, but only very rarely was there complete infiltration with involvement of the parietal peritoneum. Only in one animal was practically the entire tumour in the peritoneum. In a number of cases, small globules of tumour tissue were seen floating free in the haemorrhagic exudate. No mesotheliomas occurred in the control animals but in one of the Standard controls there was a focus of mesothelial proliferation on the serosal surface of the spleen.

The histological features of the tumours were varied and similar to those seen in man. In the majority of cases there was a mixed pattern containing both spindle-celled and epithelial elements. Most frequently, the spindle cells predominated, only occasional clefts lined by epithelial cells being present (Fig. 3). In other cases, only a few foci of epithelial cells were seen. In a smaller proportion of these tumours only an occasional area of spindle cells was observed. In some cases both patterns were widespread. As can be seen from Tables V and VI neither the pure spindle-cell nor the tubulo-papillary pattern were common. However, in both experiments the tubulo-papillary tumours were more frequent ($P < 0{\cdot}01$) for the mesotheliomas with chrysotile than with the other dusts. The spindle-celled pattern was more common with amosite in both experiments but this observation was not significant. The finding of histochemical evidence of hyaluronic acid was less frequent than expected. This was usually found when cystic tubules were present (Fig. 4), either in the pure tubulo-papillary variety or else in the mixed tumours. It was usually possible to predict the presence of hyaluronic acid on the histological appearance, particularly when the cystic dilatation of the tubules gave an appearance which superficially resembled that of fatty connective tissue. The histochemical staining was only consistently successful if the tissue had been fixed in formol-alcohol acetic acid (Tellyesniczky, 1898). In some cases, particularly with tumours of the mixed type, there was no cystic tubular tissue in the specially fixed block, so the low incidence of secreting tumours is partly explained on the failure of tissue selection.

*Injection site tumours*

As can be seen from Tables III and IV, these tumours occurred mainly in the SPF group. Only 2 were found among the Standard animals as compared to 17 with the SPF. The Standard animals were injected later and this probably indicates an improvement in technique, as their presence is an indication that the inoculum was deposited in the chest wall and not in the pleural cavity. These lesions were only seen in the animals inoculated with the amosite or the crocidolites. As described (Wagner, 1966), definite granulomata were observed in the thoracic cavity following successful intra-pleural inoculations. The presence or absence of these granulomata was recorded in all cases. This could not be done with the chrysotile as in many of the animals inoculated with this material no distinct nodules were seen. No injection site tumours were observed in animals injected with this dust. As mentioned earlier, this dust was the easiest to inject.

These tumours usually presented as large masses, superficially resembling fibroadenomata of the breast. On examination, it was found that the tumours were attached to the chest wall and were not mobile in contrast to the fibroadenomata. These tumours grew rapidly and the majority of affected animals had to be killed as they were being physically incapacitated by the size of the masses. At necropsy, the tumours were not encapsulated but were infiltrating the surrounding tissues and the intercostal spaces on the superficial aspect, only very rarely was there any penetration of the rib cage and only in one case had there been a nodule extending through the parietal pleura. If numerous slices were cut through the tumour, there was usually a small nodule, or nodules, towards the costal surface which had the macroscopic features of a crocidolite or amosite granulomata being either blue or brown in colour. Histological examination of these tumours showed the features of fibrosarcomata, usually well differentiated; occasionally there were areas of giant cell formation and occasional tumours had a pleomorphic appearance. In one or two cases, the histological appearance was suggestive of rhabdomyosarcoma, but this could not be confirmed, as no evidence of striations or myofibrils was observed after suitable staining. In all these tumours, foci containing asbestos fibres were seen. In most cases definite asbestos granulomata were seen surrounded by tumour tissue.

*Other malignancies*

These include a wide spectrum of carcinomas and sarcomas originating in practically every organ in the body. They were more common in the SPF than the Standard animals and the incidence was higher in the control groups. These findings are probably attributable to survival to an older age, the controls in both series outlived the dust-injected animals and the SPF tended to live longer than the bronchitic Standard animals.

*Survival times*

The distributions of survival times after injection are given in Fig. 5 and 6 distinguishing between animals which had a mesothelioma or injection site tumour, both conditions being the result of the injected asbestos, and animals with no such condition. The mean survival times for each sex, treatment and for animals with or without a mesothelioma and injection site tumour are given in Table VII. In Fig. 5 and 6 and Table VII, and also in the analysis to be given later, animals with no histology have been excluded except for those in the saline control group which it is assumed could not have had a mesothelioma nor injection site tumour. The number of such exclusions is small and can therefore have no important effect on any conclusions.

FIG. 5.—Distribution of survival times—SPF rats.

### ANALYSIS

Before examining treatment differences it is first necessary to check that differences between the six batches of animals and differences between the sexes may safely be ignored. For SPF rats the percentage of animals injected with asbestos which developed a mesothelioma ranged from 52 % to 60 % over the six batches. For Standard rats the corresponding range was 51 % to 68 %. The mean survival time after injection had ranges over the batches of 732 to 788 days for SPF rats and 626 to 693 days for Standard rats. Thus for both experiments the batches were reasonably homogeneous both with respect to formation of mesotheliomas and overall viability, and, as was expected, it is possible to combine the batches without loss of information.

The number of mesotheliomas showed remarkable agreement between the sexes, the 210 mesotheliomas in SPF rats being composed of 105 in each sex and the 207 mesotheliomas in Standard rats consisting of 104 in males and 103 in females. A comparison of survival times is given in Table VII. For SPF animals the mean survival times of the controls were similar for the two sexes but the mean survival time of animals developing mesotheliomas was shorter for males than females for all types of asbestos, the mean difference being 59 days. For Standard animals the male controls had a mean survival time 68 days shorter

FIG. 6.—Distribution of survival times—Standard rats.

TABLE VII.—*Mean Survival Times (Days) After Injection*

| | SPF | | | Standard | | |
|---|---|---|---|---|---|---|
| | Mesothelioma or injection site tumour | Others* | Mean | Mesothelioma or injection site tumour | Others* | Mean |
| Amosite | | | | | | |
| Males . . . | 798 | 796 | 797 | 816 | 732 | 756 |
| Females . . | 877 | 799 | 841 | 787 | 699 | 728 |
| Mean . . . | 844 | 797 | 819 | 801 | 716 | 742 |
| Chrysotile | | | | | | |
| Males . . . | 570 | 729 | 614 | 597 | 552 | 580 |
| Females . . | 633 | 697 | 660 | 639 | 528 | 612 |
| Mean . . . | 598 | 709 | 637 | 621 | 542 | 597 |
| Crocidolite | | | | | | |
| Males . . . | 701 | 630 | 677 | 652 | 598 | 635 |
| Females . . | 736 | 786 | 756 | 659 | 571 | 637 |
| Mean . . . | 718 | 715 | 717 | 655 | 587 | 636 |
| Extracted crocidolite | | | | | | |
| Males . . . | 704 | 683 | 697 | 653 | 559 | 628 |
| Females . . | 720 | 734 | 724 | 629 | 586 | 611 |
| Mean . . . | 712 | 708 | 711 | 642 | 575 | 620 |
| Saline | | | | | | |
| Males . . . | — | 889 | 889 | — | 688 | 688 |
| Females . . | — | 877 | 877 | — | 756 | 756 |
| Mean . . . | — | 883 | 883 | — | 725 | 725 |

* Animals with no histology are not included except for the saline treatment.

than the females but there were no consistent differences between the sexes in the survival of animals with mesotheliomas and, meaned over types of asbestos, these survivals were similar for males and females. Thus for both experiments differences between the sexes are small compared with the overall variations in survival times shown in Fig. 3 and 4 and also each treatment is composed of equal, or almost equal, numbers of each sex so that it is therefore valid to analyse the experiment without taking account of sex.

Two readily available indices which may be used for treatment comparisons are the proportion of animals developing mesotheliomas and the mean survival time. These will be commented on briefly although as will be shown later they are not fully informative indices. The two experiments are in general agreement on the proportion of animals with mesotheliomas. First, all types of asbestos produced mesotheliomas. Secondly, the fewest mesotheliomas were produced by amosite, 40 % of the SPF animals being affected and 31 % of the Standard animals. Thirdly, differences between the three other types of asbestos were small; the percentage of SPF animals with mesotheliomas being 64 % for chrysotile, 59 % for crocidolite and 59 % for extracted crocidolite whilst for Standard animals the corresponding percentages were 69 %, 68 % and 64 %. Comparing mean survival times for SPF the pattern is clear, the controls having a larger mean survival than any of the four asbestoses, amosite longer than the two crocidolites which were similar and in turn longer than chrysotile. For the Standard experiment the differences are smaller and the crocidolites have only slightly longer survival than chrysotile, and the controls and amosite have similar survivals.

The disadvantage of the above two indices is that both are the consequence of two factors, the first being the natural mortality experienced by a group of animals of a given age whether injected with asbestos or not, and the second the increased mortality due to the risk of developing a mesothelioma (by the use of the term *natural mortality* we here mean mortality with no condition which can be specifically related to treatment and we do not exclude the possibility that controls and treated could have different natural mortalities due to the treatment giving an increased risk of death due to non-specific causes). Comparison of both indices for a treatment group with the controls gives some indication of the risk of developing a mesothelioma and the associated increase in mortality, but does not give any indication of possible differences in natural mortality. It should be noted that the mean survival times of animals without mesotheliomas cannot be used for this purpose; *e.g.* the mean survival times of SPF animals dying without mesotheliomas is 6 months less for animals injected with chrysotile or crocidolite than the controls, but this difference could either be a real effect of treatment on mortality due to non-specific causes or it could simply be the result of the mesothelioma mortality reducing the number of long term survivors.

To overcome the above difficulties we require to separate the two types of mortality and examine the mortality with mesotheliomas eliminating mortality due to other causes. It is also of interest to examine the mortality due to other causes eliminating the mortality with mesotheliomas. The adjustment of mesothelioma mortality for natural mortality is similar to the adjustment of tumour induction rates for natural mortality in skin painting experiments and the adjustment may be performed by life table methods (Irwin and Goodman, 1946; Pike and Roe, 1963). The other adjustment is the exact parallel of the above with mortalities reversed. Mortality due to natural causes is usually considered only as a nuisance, rather than of interest in its own right, but in this case where there is a control group available for comparison, and where 40 % of asbestos injected animals died from natural causes there is some point in looking at this aspect. There is one important difference between these experiments and skin painting experiments and that is that in the latter a skin tumour may be observed as soon as it appears but in our experiments a mesothelioma is not observed until the animal dies. The effect of this delay would be to reduce the natural mortality as calculated by the above method, since at any time when mesotheliomas are being produced not all the living animals will be at risk of dying without a mesothelioma, some will already have a mesothelioma which has not yet produced any observable effect. The length of this delay is not known and in the absence of this knowledge no adjustment will be made for it.

In the analysis discussed below it is necessary to take some action on the injection site tumours. In the SPF experiment there were 17 such tumours and these occurred in animals in which the dust did not reach the pleural cavity. It is possible that in other animals the dust did not reach the pleural cavity, but an injection site tumour did not develop. Such animals may be identified by the absence of granulomata within the thoracic cavity, and this has been done for the amosite and crocidolites. There were 5 amosite, nil crocidolite and 2 extracted crocidolite animals, in addition to the animals developing injection site tumours, in which these granulomata were not observed. All these animals were excluded from the analysis so that the analysis was carried out on animals in which the dust had reached the pleural cavity. It was assumed that all the animals injected with chrysotile were in this category. In the Standard experiment there were only two such tumours and the above approach was not considered necessary; in the analysis these two tumours were combined with the mesotheliomas.

The results of the above approach using 50 day intervals are shown in Fig. 7, 8, 9, and 10. In these figures the extracted crocidolite treatment is not shown as its results were so similar to the crocidolite that its inclusion would unnecessarily complicate the figures. It is also clear from Fig. 5 and 6 and Table VII that the two crocidolites gave very similar results. Nevertheless in the analysis the two types were kept separate.

In Fig. 7 and 8, the survival of animals developing mesotheliomas eliminating other mortality is shown. For each dust the patterns were similar except for timing consisting of an initial period during which no mesotheliomas occurred and then a rapid onset of cases resulting in 50 % mortality in about the next 300 days. The survival times of the first animal dying with a mesothelioma are given in Table VIII. For SPF amosite there was a single mesothelioma occurring after 398 days,

TABLE VIII.—*Survival Time (Days) of First Animal Dying with Mesothelioma*

| | Amosite | Chrysotile | Crocidolite | Extracted crocidolite |
|---|---|---|---|---|
| SPF | 398* | 361 | 440 | 388 |
| Standard | 557 | 353 | 417 | 376 |

* This was an isolated case, the second not occurring until 666 days.

9 months before the second mesothelioma with this treatment. It is clear from Fig. 5 and 7 that this mesothelioma does not fit into the general pattern and it will not be considered further. In both experiments amosite produced mesotheliomas

FIG. 7.—Survival of SPF rats with mesotheliomas after eliminating effect of mortality due to other causes.

FIG. 8.—Survival of Standard rats with mesotheliomas after eliminating effect of mortality due to other causes.

**Fig. 9.—Survival of SPF rats without mesotheliomas after eliminating effect of mortality due to mesotheliomas.**

**Fig. 10.—Survival of Standard rats without mesotheliomas after eliminating effect of mortality due to mesotheliomas.**

much later than the other treatments and crocidolite later than chrysotile, but in the Standard experiment the latter difference was only of the order of 20 days.

Fig. 9 and 10 are the corresponding figures for natural mortality. In the SPF experiment apart from some excess deaths from chrysotile between 700 and 750 days there are no appreciable differences between the treatments and control. In the Standard experiment differences are again small and the only consistent pattern is that the controls showed a higher mortality than the treated. As discussed earlier such a result might be expected and be an artifact due to the delay in a mesothelioma causing death. Since this effect was not found in the SPF experiment and is small in the Standard experiment it is suggested that the bias caused by ignoring the delay is not large.

The results of these experiments are clear cut and no further analysis is necessary. However the model relating induction rate of tumours with time discussed by Pike (1966) has been shown to summarise the data adequately (Berry and Wagner, 1969). The establishment of the validity of this approach on these two large experiments serves as justification for following it in the analysis of similar experiments where it is necessary to perform a more formal analysis.

### DISCUSSION

The analysis of these two experiments by separating the two types of mortality, the one due to causes definitely attributable to the treatment and the other to causes not so attributable, has given a simple summary of the data, in spite of the complication that a mesothelioma does not result in immediate death. With regard to this complication there was a discrepancy between the two experiments in that in the Standard experiment the calculated natural death rates of treated animals were less than for the control animals, an expected artifact, but this was not found in the SPF animals.

In SPF animals in which the dust did not reach the pleural cavity, there was a high incidence of injection site tumours, 5 out of 10 for amosite, 4 out of 4 for crocidolite and 8 out of 10 for extracted crocidolite.

The mesotheliomas were similar to those seen in human cases and cover the same spectrum of histological patterns. The presence or absence of infection does not appear to have materially affected the incidence of tumour development. Both crocidolite specimens produced a large number of tumours. The amosite produced fewer tumours in both experiments, the period between inoculation and tumour development being longer than with the two other types of asbestos. There was a similar number of mesotheliomas with chrysotile as with the crocidolites, but the tumours occurred slightly earlier.

The high incidence of these neoplasms following the inoculation of chrysotile was unexpected. The chrysotile used was from a specially prepared batch that had been produced by a sedimentation process from the lowest commercial grade of fibre. The possibility that it may have contained some contaminant has been investigated in laboratories in Britain and the United States, and nothing has been found which is significantly different from other chrysotile samples (Morgan, 1968 and Cralley, 1968, both personal communications).

In a later experiment animals have been inoculated intra-pleurally with a number of chrysotile samples from different sources including the chrysotile used in these experiments. The results of this study will show whether the presented findings are typical for this variety of fibre.

The significance of the presence of oils and waxes associated with the various types of asbestos requires further investigation. In this experiment two preparations of the crocidolite sample were used. From one preparation, Harington attempted to remove all the oils and waxes by repeated reflux extraction in cyclohexane, until there was no evidence of fluorescence in the solvent when examined in UV light. The other was untreated and contained the contaminating hydrocarbons. As can be seen from the results, the presence or absence of the oils has not affected the incidence of tumour production. However, further studies by Harington and Commins (1966, personal communication) have shown that the cyclohexane extraction does not remove all the oils, this requires the use of a series of solvents. In addition, they showed that asbestos fibres would adsorb oils from hessian and jute sacks. Recently, Commins and Gibbs (1969) have shown that asbestos is a powerful catalyst in the formation of tetratertiary butyl diphenoquinone from the anti-oxidants in some plastics. Our samples were all stored in plastic bags and Commins found the dust to be contaminated. This finding occurred 5 years after the inoculations, so it is not possible to estimate the actual amount of contamination present at that time.

A number of other features in these studies were considered to require additional investigations, some of which have been undertaken and it is hoped to submit the findings for publication at a later date. One of these concerns the dosage; 20 mg. of dust in the inoculum may be considered too high an amount. In order to see if a dose response relationship can be established animals have been injected with crocidolite and chrysotile in doses covering the range 0·5 mg. to 8 mg.

To obtain results which can be compared with those of other investigations, the experiments have been repeated on SPF rats using the UICC Asbestos Reference Samples (Timbrell, Gilson and Webster, 1968). As an extension of this experiment, an attempt has been made to clarify the significance of the presence of oils, both natural and acquired, in relation to asbestos dust exposure. Dr. Commins has removed the oils from small amounts of the Reference Samples by repeated extractions in a variety of solvents. The oil-free material was kept in glass containers so eliminating the possibility of contamination from plastic bags. These materials have been inoculated into further groups of animals.

As mentioned previously (Wagner 1966, 1969), the whole concept of the intra-pleural inoculation of asbestos is unrealistic when compared with human experience and results obtained by inhalation would be more valid. Rats have recently been exposed to dust clouds of the Reference Samples. The results of these investigations will not be available for another 2 years.

### SUMMARY

SPF and Standard rats were inoculated intrapleurally with samples of amosite, chrysotile, crocidolite (natural and with the oils extracted) and saline. With all types of asbestos an appreciable proportion of animals developed a mesothelioma but none of the saline controls developed such a tumour. The histological features of these tumours are described. The survival times have been analysed by sub-dividing the mortality into two independent components, one due to mesotheliomas and the other to natural causes. For all types of asbestos there was a rapid onset of mesotheliomas after an initial period during which none occurred, but this initial period was dependent on the type of asbestos, being longer for amosite than for chrysotile or crocidolite. No evidence was provided of any difference in effect between the natural and oil extracted forms of crocidolite.

We wish to acknowledge the great assistance given by members of the Pathology and Physics Division of this Unit. The electron-micrographs were taken by Dr. F. D. Pooley of the Department of Mining Engineering of the University College of South Wales and Monmouthshire.

### REFERENCES

Berry, G. and Wagner, J. C.—(1969) *Br. J. Cancer*, **23**, 582.

Commins, B. T. and Gibbs, G. W.—(1969) *Br. J. Cancer*, **23**, 358.

Gilson, J. C.—(1966) *Trans. Soc. occup. Med.*, **16**, 62.

Harington, J. S.—(1962) *Nature, Lond.*, **193**, 43.—(1965) *Ann. N.Y. Acad. Sci.*, **132**, 31.

Irwin, J. O. and Goodman, N.—(1946) *J. Hyg., Camb.*, **44**, 362.

Pike, M. C.—(1966) *Biometrics*, **22**, 142.

Pike, M. C. and Roe, F. J. C.—(1963) *Br. J. Cancer*, **17**, 605.

Roe, F. J. C., Carter, R. L., Walters, M. A. and Harington, J. S.—(1967) *Int. J. Cancer*, **2**, 628.

Smith, W. E., Miller, L., Churg, J. and Selikoff, I. J.—(1965) *J. Mt Sinai Hosp.*, **32**, 1.

Tellyesnickzky, K.—(1898) *Arch. mikrosk. Anat. EntwMech.*, **52**, 202.

Timbrell, V., Gilson, J. C. and Webster, I.—(1968) *Int. J. Cancer*, **3**, 406.

Wagner, J. C.—(1962) *Nature, Lond.*, **196**, 180.

Wagner, J. C.—(1966) in ' Lung tumours in animals ', Edited by L. Severi. Proceedings of the Third Quadrennial International Conference on Cancer, Perugia, June 24–29, 1965, pp. 589–606, Perugia, Division of Cancer Research, University of Perugia, Italy, 1966.

Wagner, J. C.—(1969) in Proceedings of the Second International Conference on the Biological Effects of Asbestos, Dresden, April 22–25, 1968. To be published.

Wagner, J. C., Munday, D. E. and Harington, J. S.—(1962) *J. Path. Bact.*, **84**, 73.